# An effectiveness analysis of healthcare systems using a systems theoretic approach

**DOI:** 10.1186/1472-6963-9-195

**Published:** 2009-10-24

**Authors:** Sheuwen Chuang, Kerry Inder

**Affiliations:** 1 Health Services Research Group, University of Newcastle, 3^rd ^floor, David Maddison Building, Cnr King and Watt St, Newcastle, NSW, 2300, Australia; 2 Centre for Clinical Epidemiology & Biostatistics, University of Newcastle, 3^rd ^floor, David Maddison Building, Cnr King and Watt St, Newcastle, NSW, 2300, Australia

## Abstract

**Background:**

The use of accreditation and quality measurement and reporting to improve healthcare quality and patient safety has been widespread across many countries. A review of the literature reveals no association between the accreditation system and the quality measurement and reporting systems, even when hospital compliance with these systems is satisfactory. Improvement of health care outcomes needs to be based on an appreciation of the whole system that contributes to those outcomes. The research literature currently lacks an appropriate analysis and is fragmented among activities. This paper aims to propose an integrated research model of these two systems and to demonstrate the usefulness of the resulting model for strategic research planning.

**Methods/design:**

To achieve these aims, a systematic integration of the healthcare accreditation and quality measurement/reporting systems is structured hierarchically. A holistic systems relationship model of the administration segment is developed to act as an investigation framework. A literature-based empirical study is used to validate the proposed relationships derived from the model. Australian experiences are used as evidence for the system effectiveness analysis and design base for an adaptive-control study proposal to show the usefulness of the system model for guiding strategic research.

**Results:**

Three basic relationships were revealed and validated from the research literature. The systemic weaknesses of the accreditation system and quality measurement/reporting system from a system flow perspective were examined. The approach provides a system thinking structure to assist the design of quality improvement strategies. The proposed model discovers a fourth implicit relationship, a feedback between quality performance reporting components and choice of accreditation components that is likely to play an important role in health care outcomes. An example involving accreditation surveyors is developed that provides a systematic search for improving the impact of accreditation on quality of care and hence on the accreditation/performance correlation.

**Conclusion:**

There is clear value in developing a theoretical systems approach to achieving quality in health care. The introduction of the systematic surveyor-based search for improvements creates an adaptive-control system to optimize health care quality. It is hoped that these outcomes will stimulate further research in the development of strategic planning using systems theoretic approach for the improvement of quality in health care.

## Background

The use of accreditation systems to improve healthcare quality and patient safety has been widespread across many countries [1-4]. Quality measurement incorporating clinical indicators and quality indicators, and reporting systems, have grown substantially as the more visible aspects of hospitals' quality improvement efforts [5-9]. Taken together, these systems comprise the health administration segment of the healthcare system, for convenience labelled the health administration system. The health administration system is believed to influence quality outcomes and considerable resources are spent by participating hospitals in this belief.

There is rich research literature on the association of the accreditation and measurement/reporting systems to quality in health care, but the results are unsatisfactory. The outcome of quality is not well correlated with accreditation requirements, even when hospital compliance with accreditation and measurement/reporting requirements is acceptable. In general, partial, inconsistent or conflicting results have been discovered [7,10]. An important feature of this research is that it is concerned only with correlation, rather than the processes through which the impact of the systems occurs, and is fragmented: specialized to specific clinical or management perspectives or a system or subsystem taken in isolation. The fragmented research on the determinants of quality in healthcare reveals partial observation and ambiguous results.

Owing to these findings, some arguments have been made for "a more systematic use of theories in planning and evaluating quality-improvement activities in clinical practice" [11-13]. The idea is to use theories to describe the model lying behind a specific intervention and then design research to evaluate the model. The need for a theoretically driven approach to understanding complex social interventions and their effects has been strongly advocated [14] as the way to gain knowledge about the overall systemic effects of the health administration segment acting on the health care system, especially knowledge that will inform decisions about the use of health care resources to support the most valued processes. Yet, for all the interest in the use of the accreditation and measurement/reporting systems to improve healthcare quality and patient safety, the science of healthcare performance measurement and management is still relatively embryonic, and there remains a paucity of hard evidence to guide policy and research planning from a firm theoretical basis.

According to systems theory, patient safety and quality of healthcare is an emergent property of the entire healthcare system [14], it follows that the improvement of health care outcomes needs to be based in a systematic appreciation of the whole system that contributes to those outcomes. Yet the research literature currently lacks an appropriate analysis of this sort. Therefore, the first aim of this paper is to use systems theoretic approach to develop an integrated research model of the accreditation and quality measurement/reporting systems, taken in relation to the hospital-level healthcare system. The second aim is to demonstrate the usefulness of the resulting model for strategic research planning. The paper provides an example of an adaptive-control study derived from the proposed model. It demonstrates a template for more advanced strategic research planning of quality improvement throughout the healthcare systems.

## Methods/design

To achieve these aims, the research was conducted through the combination of a theoretical based study and a literature-based empirical study. The theoretical based study combines the basic concepts of systems theory and a general systems flow with the Supply Input Process Output Key Stakeholder (SIPOKS) process model to form the systems theoretic approach. The approach is used to initially develop a basic high level integrated systems model as a framework to guide the investigation of the effect of the accreditation and measurement/reporting systems on health care quality and then to examine, from a systems flow perspective in a lower level, the causal links within the system that impact on its effectiveness.

A literature-based empirical study used existing research or documentation as evidence with which to validate the proposed general relationships derived from the model. Australian experiences in accreditation and clinical performance data reporting, especially from the Australian Commission on Safety and Quality in Health Care and the Australian Council on Healthcare Standards (ACHS), are used as the major evidence base for evaluating the performance of these relationships and further as a design base to develop an example of an adaptive-control study. In order to gain a better understanding of systems theory, healthcare systems hierarchy, general systems flow and the SIPOKS process model, and the Australian research base used to form the adaptive-control study, their use is elaborated below.

### Systems theory

The foundation of systems theory rests on two pairs of concepts: emergence and hierarchy; control and communication [14,15]. According to the first pair of systems theory concepts, a general model of complex systems can be expressed in terms of a hierarchy of levels of organization [16]. The safety and quality characteristics of complex systems are an emergent property of the system as a whole, not a property of individual system components. According to the second pair of basic system theory concepts, an open and dynamic complex system like the healthcare system is viewed as a suite of interrelated subsystems that are kept in a state of dynamic equilibrium by feedback loops of information and control [17]. Specifically, their relevant emergent properties are controlled by a set of safety and quality constraints related to the behavior of the system components or subsystems [18]. Regulation to required standards is the common form that enforcement of safety constraints takes in complex systems and is expressed through hierarchical regulation relationships [19]. Since control implies the need for communication, reverse communication within the system hierarchy from controlled to controller is required to stimulate systems' behavior towards the accepted standard of safety and quality [20].

In the study, systems theory is applied to construct a healthcare system hierarchy which consists of interacted systems linked with control and communication in different layers.

### Healthcare systems hierarchy

**Figure 1:**
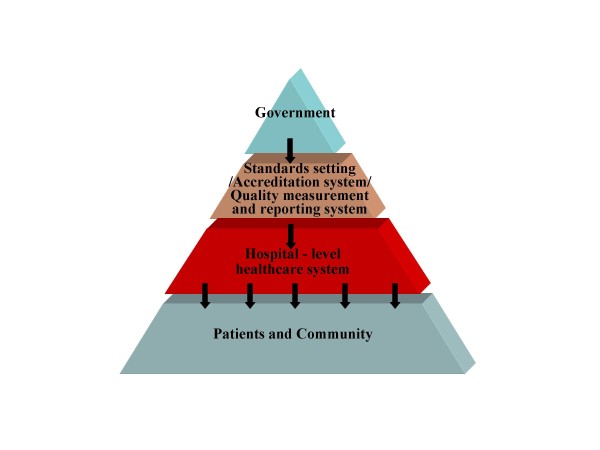
**Healthcare Systems Hierarchy**.

For the purposes of this paper the overall healthcare system may be simplified to a 4-layer model shown in Figure 1. Inter-layer relationships are characterised by vertical control and communication, but the full regulatory structure also includes significant horizontal interrelations as well as self-regulation. The proper functioning of all these relationships is important to the ultimate achievement of quality health care [19]. The focus of this paper is on the middle two layers, the health administration system, with its two sub-systems - the accreditation system and the quality measurement and reporting systems - in relation to the hospital-level healthcare system. A holistic healthcare systems relationship model made of the two-layer system hierarchy is constructed for detailed analysis. The relationship of the two health administration sub-systems to one another and to the hospital-level healthcare system 'below' them forms the focus of this study.

### General systems flow and SIPOKS process model

A general systems flow is used where systems receive inputs and utilise, transform and otherwise act on them to create outputs, whether to other systems or the external environment [21]. The SIPOKS process model partitions the overall systems flow into Suppliers, Input, Process, Output, and Key Stakeholders for convenient analysis. In this model, the supplier provides the required inputs to a system process, including people, equipment, materials, working procedures and methods, and general working environment. The process then utilises, transforms and otherwise acts on the inputs to produce a set of outputs that are used by key stakeholders, which may be suppliers to other processes in the same or different systems. A stakeholder is defined as any group that is impacted or interested in the performance of the process and the word 'key' denotes important stakeholders. Applied to each of several interrelated sub-systems, SIPOKS can, for example, usefully analyse an extended process into shorter phases and link the analysis of interacting processes from different hierarchical levels for a specific purpose [22]. In this way, the SIPOKS analysis can provide insight into cause-effect relationships within systems.

A literature-based empirical study used a wide range of existing research or documentation, from several countries, concerning the functioning of these systems and their interrelations as a basis for imputing general flow processes to them, of the hierarchical control and communication type which were of interest to the safety and quality of health care. The model processes obtained were thus broadly validated by their occurrence throughout developed healthcare systems. Subsequently, the more detailed Australian data were used to evaluate the performance of the key system interrelationships, as reported below.

### The Australian research base

The Australian Council on Healthcare Standards (ACHS) was the first in the world to introduce clinical indicators as part of the health care accreditation process. Sixty three percent of all Australian public hospitals and 74% of private acute and psychiatric hospitals are ACHS accredited [23]. Six hundred and eighty nine hospitals reported clinical indicators in 2007, which covers all Australian states and territories and also New Zealand. Data from public (49%) and private (51%), metropolitan and non-metropolitan hospitals are included [24]. More recently, the ACHS has moved accreditation into the era of continuous quality improvement as reflected in the Evaluation and Quality Improvement (EQuIP) standard. The target of the more holistic accreditation process required by EQuIP-4 is to have accredited organizations embrace the continuum of ways to improve healthcare safety and quality in Australia [25]. The study example proposed in this paper is based on the ACHS standards and data and is intended to contribute a research template for promoting continuous improvement in health care.

## Results

Using a systems theoretic approach, the holistic healthcare systems relationship model was developed, the overall effectiveness of the accreditation and measurement/reporting systems for providing quality of care was identified, and system weaknesses from a system flow perspective were discovered. An example of an adaptive-control study involving accreditation surveyors derived from this approach is developed.

### The holistic healthcare systems relationship model

**Figure 2:**
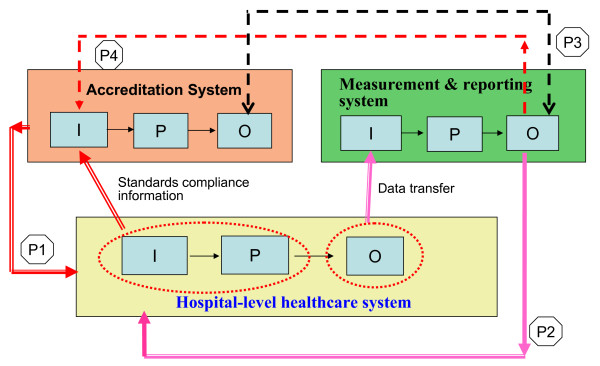
**Holistic Healthcare Systems Relationship Model**. P1: focus on control relationship with communication. P2: focus on communication without control. P3: concern with communication (in general, no association found, cannot communicate each other). P4: intend to be as a control relationship (to increase the control efficacy of accreditation system on hospital and communication efficacy of quality measurement/reporting system with hospital)

In practice, the hospital-level healthcare system is typically impacted by numerous accreditation and clinical or quality performance reporting systems, typically clinically differentiated. Ultimately these systems need to be discriminated and treated individually; however, in this study only their overall, shared interrelations are considered. Combining the concept of healthcare systems hierarchy and an analysis of control and communication relationships, a basic holistic healthcare systems relationship model is designed as shown in Figure 2. As both the horizontal and vertical control/communication relationships are potentially relevant to maintaining an acceptable level of quality within the healthcare systems hierarchy, there are in principle four model relationships of direct interest. These are labeled P1 to P4 in Figure 2. Of note, the fourth relationship (P4) has hitherto essentially gone unrecognized and its potential remains underdeveloped. It will figure prominently in the study design proposal.

Two of these four relationships (P1 and P2) are vertical control and communication relationships, providing the outputs of the accreditation and the quality measurement/reporting systems, respectively, as inputs to the hospital-level healthcare system to improve quality of care. Relationship P1 is a control relationship that provides hierarchically determined practitioner standards while the P2 communicates outcomes to the hospital-level healthcare system for its own internal control response.

The remaining two relations, P3 and P4, are horizontal control and communication relationships within the health administration system, to improve the focus and impact of accreditation on quality of care. P3 communicates correlations in the outputs of the accreditation and the quality measurement/reporting systems and P4 provides feedback from the output of the quality measurement/reporting system to the accreditation system input. While P3 is concerned with communication, P4 is intended as a control relationship. Strictly, P3 is at present a quasi-relationship because in practice there is no specific recipient of this research-generated information within the health care system. The information is in effect an indicator of system-wide coherence, and in that sense relevant to all, however no-one has a mandate to respond to it. In a more systematic institutional design this information might be fed to a government source (top layer of Figure 1) or other system-wide responsible entity to initiate and focus health care improvement research of the kind proposed below. This is a potentially important relationship outside the scope of this paper. Or it might also be directly utilised by P4 to focus its feedback as proposed here. In what follows it is treated as a potential communication relationship.

With respect to each of these relationships, the question arises as to what is known about its impact on quality of care. While considerable research has been directed toward understanding and improving P1 and P2 to produce improved health care, only P1 is well understood and supports practical reform. P2 has no satisfactory outcome as discussed below under 'Effectiveness of quality measurement and reporting system'. As for P3 and P4, complex, ambivalent findings hold in P3 and it currently lacks an effective feedback role, while little or no research has been directed toward understanding and improving P4. These claims are reviewed below for the four relationships.

### Effectiveness of the accreditation system

The evaluation of P1 assesses the effectiveness of the accreditation system for providing quality of care. Comparative effects on hospitals during and after the implementation of an accreditation process have been evaluated. Studies have found that accreditation systems provided better quality results in nursing care [26]; positive changes in leadership, commitment and support, use of data, staff involvement, and quality management [27]; improved outcomes in trauma health care [28]; and the enhancement of patient care through organizational strategies introduced as a result of participating in an accreditation program [29]. However, there are other studies which did not support these findings [30,31].

In the Greenfield and Braithwaite review [10], ten categories were identified for research into the impact or effectiveness of health sector accreditation. The categories of promoting change and professional development provided consistently positive findings, however findings were inconsistent in five categories: professions' attitudes to accreditation, organizational impact, financial impact, quality measures and program assessment. The small numbers in the remaining three categories (consumer views or patient satisfaction, public disclosure and surveyor issues) precluded any valid conclusions. These outcomes support the view that the relationship between the accreditation system and hospital-level healthcare system has at least some positive impact.

The reports of the Australian Commission on Safety and Quality in Health Care discussed the effectiveness of accreditation processes and contained feedback from consultation with focus groups and stakeholders [32,33]. This feedback has been analysed using the SIPOKS model to understand the systemic weaknesses involved from a system flow perspective, as shown in Table 1. Identified weaknesses related to each issue are recognised by a check mark in the corresponding field.

**Table 1:** SIPOKS analysis of accreditation system issues for system flow weakness

**NO**.	**Accreditation System Issues**	**Key Stakeholder (KS) feedback**	**O**	**P**	**I**	**S**
1	Effectiveness of the accreditation system to detect poor performance	Divergence of views in relation to the capacity and appropriateness of accreditation for detecting poor performance		x	x	

2	Transparency of information on the accreditation status and quality of health services	Many stakeholders sought open access to accreditation outcomes and the decision making process of accreditation bodies. Some stakeholders have counter arguments	x	x		

3	The separation of standard setting and accrediting functions	Opinion is divided on whether this would result in overall systems benefits or losses				x

4	Duplication and overlap in the accreditation system	Significant consensus	x	x	x	x

5	Resources investment in accreditation is disproportional to the gains	Significant consensus	x	x	x	x

6	Inter and intra surveyors' reliability, availability and sustainability	Issues are acknowledged by submissions and focus group			x	x

7	The use of accreditation outcome at a national level to support policy and prioritisation of safety and quality	The timeliness, accuracy, reliability and validity of the data were all questioned by stakeholders	x			

8	Proliferation of standards results in time and resources consumed	Significant consensus			x	x

9	Access to Standards in a user friendly format	It is a cost issue				x

Stakeholder Consultation Statistics:		Written submission/Focus group participants				
• Private health service or Not for profit health service		(15)/(72)				
• Public Health Service		(6)/(113)				
• Other Government Bodies		(4)/(54)				
• Professional or Member Organisation		(34)/(9)				
• Accrediting or Certifying Bodies		(6)/(37)				
• Standards Bodies		(2)/(21)				
• Individuals or Quality or Complaints Bodies		(13)/(25)				

**NOTE**: SIPOKS is reversed from left to right. O: output, P: process, I: input, S: supplier

X means the segment of system flow is related to the issue

According to the results of this SIPOKS analysis, weaknesses are spread broadly across the categories: suppliers, inputs, processes, and outputs. Most accreditation system issues come from supplier and input problems. Suppliers are the providers of the required inputs to the accreditation processes, such as the standard setting bodies and hospitals providing surveyors. Inputs are used in the accreditation processes, and include surveyors, survey methods or procedures, and survey standards. The present unsystematic variety of accreditation bodies and standards has resulted in unhelpful complexity, increased time and manpower costs, and variable reliability of surveyors. Although relationship P1 has shown some positive impact on quality of care, the future reform of the accreditation system could consider enhancing the effectiveness for providing quality of care from a system flow perspective, and establish an adequate control between accreditation and hospital-level healthcare system.

### Effectiveness of quality measurement and reporting system

The evaluation of the P2 relationship assesses the effectiveness of the quality measurement and reporting system for improving the quality of care. Again, research has identified some issues concerning the quality of measurement and reporting systems, principally concerning data validity, reliability, timeliness, and meaningfulness [34-38]. These issues were analysed using the SIPOKS model to identify weaknesses from the system flow perspective, see Table 2.

**Table 2:** SIPOKS analysis of quality reporting systems issues for system flow weakness

**NO**.	**Public Reporting Issues**	**Key Stakeholder (KS) Concerns**	**O**	**P**	**I**	**S**
1	Accurate and complete source data	Accuracy of pre-populated administrative data, Abstraction inconsistent with abstraction guidelines, Week abstraction guidelines and/or data element definitions, Technical issues			x	x

2	Inconsistencies among various data sources for varied reporting systems	Problems with the universality of coverage and data standardization, Inadequacy of existing IT systems, manpower consumed			x	x

3	Validity and meaningfulness of output data, provision of performance benchmarks	Hospitals and physicians distrust and attempt to discredit the data. Benchmarking and interpretability needs to be enhanced	x	x		

4	Minimal random error	Appropriate statistical processing		x		

5	Accessibility and Awareness of all levels of staff	Not all levels of medical staff can access the data, misunderstanding of data	x			

6	Timeliness	More of historic interest than a means of identifying present-day care, the change had already occurred by the time the data were issued.	x	x	x	x

7	Supplementary quality improvement support by reporting systems	Training and other quality improvement support can attract the involvement of hospitals	x			

**NOTE**: SIPOKS is reversed from left to right. O: output, P: process, I: input, S: supplier

X means the segment of system flow is related to the issue

Most of the system weaknesses concern either input or output. Imperfections in input data, input technique, and the motivation of internal reviewing and auditing of data in hospitals, cause the problems of data validity and reliability [37,39]. Problems with the timeliness, validity and meaningfulness of output data are associated with the whole system flow from suppliers, to input, process, and output to key stakeholders. Here the suppliers are the hospitals who provide the data. Hospitals also are one of the key stakeholders who use the analysis report. To increase the quality of feedback and the utilization of performance reports, we need efforts from both hospitals and the report processing agencies in the system flow perspective.

Under the present conditions, studies of the efficacy of quality measurement and reporting systems in stimulating quality improvement show mixed results. Some quality measures have been shown to have a positive impact on quality improvement [6,40-42]. In particular, the reporting systems linked to payment, accreditation, and peer pressure from public benchmarking have made quality measurement and improvement higher priorities for hospital leadership and achieved better results [42]. Some conflicting findings show no effect on quality results [43-45]. From this evidence, we may conclude that besides the system weaknesses of quality measurement and reporting system itself, the impetus from external systems can stimulate positive results.

### Association between the two administrative systems' outputs

The evaluation of the P3 relationship assesses the coherence between the accreditation system and the quality measurement and reporting system. Although some studies found that quality measures have been shown to improve care outcomes in health organisations [46,47], no simple overall relationship is generally found between accreditation outcomes and quality performance measures [10,48,49]. The research examined the Australian EQuIP standards and Australasian Clinical Indicator Report [24,25] using the systems theoretic approach to explain their relationship.

Most of the EQuIP accreditation standards required to be complied with by hospitals belongs to the structure and process elements of the accredited agency. The structure elements in system flow perspective are the elements of input of the hospital-level healthcare system. Therefore, it is identified in Figure 2 that the segment of input and process in the hospital-level healthcare system provide the standards compliance information to accreditation system. Each clinical indicator in the quality measurement and reporting system is either a process indicator, measuring the activities and tasks in patient episodes of care, or an outcome indictor, measuring the effects of care on the health status of patients and populations, which is the final result of all related processes [50]. The two kinds of clinical indicators belong to the category of output in the system flow perspective. It is identified in Figure 2 that the segment of output in the hospital-level healthcare system provides indicators data to accreditation system.

Figure 2 displays clearly that the accreditation system and the quality measurement and reporting system are not concerned with the same target (system segment). In addition, the evaluation of some inputs and processes during accreditation cannot encompass the whole range of characteristics constituting the emergent quality and patient safety output of the overall system, nor are these fully measured just by the partial clinical indictors currently in use. Indeed, these gaps leave open the possibility that these two outputs are sometimes associated by chance. From this systems perspective we can thus understand why quality indicators often appear unrelated to accreditation outcomes [48,49], yet some association between these two systems' outputs has also been found [46,47]. To make the outputs of the accreditation and measurement/reporting systems coherent, that is, fully correlated, it is necessary to systematically investigate both the quality consequences of deliberate changes in the elements of the accreditation process, such as accreditation methods and training of surveyors, and of the specification or calculating algorithms of quality measurement in the reporting system. The proposed adaptive-control study flows from this conclusion.

### Interactive effects between the two administrative systems

For systems that are designed independently it is unusual to find significant coherence between their outputs. This is essentially what has happened with the accreditation and measurement/reporting systems. The systems flow analysis suggests a way to remedy this historical defect: use the fact that P3 is a communication relationship to locate the corresponding control relationship, P4, and exploit its capacity. This relationship is currently unrecognized and is arguably central to a overcoming the present fragmented research on improving the impact of accreditation on quality of care and hence on accreditation/performance coherence (correlation).

Evaluating the potential of the P4 relationship is assessing the potential inherent interactions between the accreditation and measurement/reporting systems and the extent to which their effects could be expected to impact on the control and communication process between these two horizontal systems. In this respect it is noted that the enhancement of the accreditation standards to demonstrate continuous quality improvement in organizational and clinical practice has been requested by the accreditation body as a way of ensuring consistency between the accreditation evaluation and positive quality improvement in health care [25].

Recall that the required inputs to the accreditation process principally include standards and surveyors. Moreover, surveyors could be one of the key stakeholders of the quality measurement and reporting system. They compare hospital services against best practice encapsulated in standards developed and agreed by professionals and users. They are encouraged to carry out follow-up surveys to maintain continuity. However, based on limited research of accreditation surveyors, surveyors' current practices revel that their actions have substantial impacts on accreditation but carry potentially important limitations on quality improvement [51-53]

It is important that surveyors interpret the standards and clinical performance trends accurately and consistently to ensure both compliance and improvement by stimulating positive and longitudinal changes. If accreditation is to effectively improve health outcomes, the critical areas of concern about quality and safety in the accreditation process need to be identified correctly and investigated. This is precisely what the P4 relationship in principle permits. In summary, the systems modelling and evaluation uncovered an implicit relationship conveyed through surveyors within the health administration system, which can provide feedback between quality performance reporting components and choice of accreditation components. While this relationship is currently unrecognized, it is arguably central to overcoming the present fragmented system functioning and coherence, and through systematic research for improving the impact of accreditation on quality of care and hence on accreditation/performance correlation.

## Discussion

### A Prospective Study

The preceding analysis of the systemic weaknesses and potential of the health administration system, guides the construction of advanced, integrated research into ameliorating those weaknesses. An adaptive-control study derived from the implicit P4 relationship between the health administration systems is outlined as an example below. An adaptive-control study refers to a potentially existing situation, like the unrecognised P4 relationship. It explores what is required to implement a feedback architecture which can be adapted as we build adequate control and communication between systems for the efficient and effective improvement of quality of care.

The study objective is to understand and utilise the potential for feedback between quality performance reporting components and choice of accreditation components through the activities of surveyors within the health administration system. Surveyors are already encouraged to carry out follow-up surveys to maintain the continuity of quality improvement in health care. The study assumes that if quality performance reports had been utilized by hospitals as quality improvement guidance and referenced by surveyors as a tool to trace quality improvement, then surveyors could produce valuable feedback within the health administration system for the accreditation process.

There would be two stages to this study. Firstly, ACHS surveyors would be surveyed through a semi-structured questionnaire to gain an understanding of the current status of their utilisation of quality performance reports (six-monthly report and trend report), decision making for selection of investigation targets and ways in which health services agencies currently use the performance reports. Secondly, the association between accreditation standards and clinical indicators will be examined in detail to explore characteristics of clinical indicators that can best be used to inform accreditation processes.

The result of health administration systems is a surveyor-carried systematic search for adapting characteristics of the measurement/reporting system output so as to best influence the accreditation process in ways that best correlate to improved safety and quality of care. This has the effect of transforming the overall systems process (relationships P1 - P4) from a partial and fragmented historical construction into a systematic adaptive-control process for optimising safety and quality of care. In this sense this proposal is a prototype of all subsequent systematic research of its kind. The results of this adaptive-control study would be used to improve the design of surveyors' training material, the redesign of quality measurement and reporting system, and to create an effective and efficient mechanism for the interaction between the accreditation system and quality measurement/reporting system. In this process, current defects in the vertical P1 and P2 relationships would also be repaired.

### More system theoretic thinking in research design

Different research designs or approaches have been proposed to resolve issues of the accreditation and the quality measurement and reporting system separately. Braithwaite, et al. [54] apply multi-method approaches to focus on two central aims: to examine the relationships between accreditation status and processes, and the clinical performance and culture of healthcare organization; to examine the influence of accreditation surveyors and the effect of accreditation surveyors on their own health organisations. Joly et al. [55] propose a logic model approach which focuses on inputs, strategies, outputs, and multiple level outcomes, with emphasis on accredited public health agencies as the input of interest. However these approaches need to be complemented by research using systems theoretic approach to help address the weaknesses from a system flow perspective and to understand quality of care as an emergent whole-system property.

The quality measurement and reporting system is playing an increasingly important role in the healthcare systems. It has been utilized as a potentially complementary tool to accreditation for improving quality. However, the available evidence suggest that both the weakness of quality measurement and reporting system and the impetus from external systems to stimulate improvement can influence the effectiveness of quality of care. Therefore, improving the effectiveness of P2 requires an increase in the quality of feedback and the utilization of performance reports for data accuracy, validity, meaningfulness, timeliness, and the right stimulation from external systems, like government. This invites the systems theoretic approach to design a series of systematic studies covering each weakness from the system flow perspective and cascading up and down external systems to establish adequate control/communication relationships between systems. If we facilitate two pairs of basic systems theory concepts and system flow perspective in our research, the quality measurement and reporting system can be enhanced to support the right information in the right time for quality improvement in health care.

### Complete and adequate control and communication in healthcare systems

The research simplifies the healthcare systems into 4-layer model (Figure 1). The focus of this paper is on the middle two layers. However, it does not mean the effectiveness of healthcare systems for providing quality can be improved only through better control and communication within the two layers. On the contrary, without complete and adequate control and communication in the whole healthcare systems hierarchy, it is difficult to achieve a cost-effective emergent property and provide value for patients. Michael Porter [56] advocates that solutions tend to target one or two aspects of system for improving quality, cost and accessibility in health care have not worked, or cannot work. The nature of health care delivery needed to be transformed. The fundamental reform is to create value-based competition on results through all efforts from health care providers, health plans, suppliers, consumers, employers, and government. It needs to have one who had to transform the strategies, organizational structure, pricing approaches, and the measurement practices of the various actors in the system.

Reform must focus on how to get competition right and how to put in place the enabling conditions, such as the right information, the right incentives and time horizons, and the right mind-sets [56]. The research approach for the effectiveness analysis of the middle two-layer subsystems in Figure 1 can be used as an example for the effectiveness analysis of the whole healthcare systems hierarchy. Using systems theoretic model explores the relationships between systems and assesses their effectiveness in a clear system flow thinking. The overall analysis results of the approach provide an integrated conceptual cascade effect, which might bring possible adaptive-control studies from the additional implicit relationships between systems, or potential feasible ways of achieving the complete and adequate control/communication to create value based competition in healthcare systems could be realized more.

To improve the overall performance of quality in healthcare systems, researcher needs to adopt system theoretic thinking in research design and a holistic view of systems effectiveness. However, systems thinking can be challenging, especially taking a broad view of systems. This may limit the application of the systems theoretic approach.

## Conclusion

As safety and quality is an emergent property of the healthcare system as a whole, not a property of individual system components or subsystems, the assessment of safety and quality from any perspective in one system or using any one tool is unlikely to give the complete picture. A systems theoretic approach supported by research evidence provides the necessary holistic insight to understand the overall relationship and effectiveness among the three systems. A systems analysis reveals four inter-system relationships, the fourth hitherto unreported, along with the unsatisfactory vertical control and communication between the quality measurement/reporting system and hospital-level healthcare systems, and little or no concrete horizontal control and communication between the accreditation system and the measurement/reporting system. Overall the health administration systems do not yet have significant positive impact on the quality of care.

To help advance the science of safety and quality improvement in healthcare systems and to inform decisions on the use of healthcare resources for optimized results, the paper examines system issues using the system flow SIPOKS model to give more supporting information on the system weaknesses. It provides a system thinking structure to assist the design of quality improvement strategies. The internal structure of Tables 1 and 2, allow design of research for problems in specific segments of the system flow in an adaptive-control manner for quality improvement, phase by phase, promoting the effectiveness of system integration. An example of adaptive-control study design is derived from the implicit P4 relationship between the health administration systems, that can overcome the present fragmented state of communication and control relationships among the relevant systems.

The effectiveness of quality delivered by each subsystem in the healthcare systems hierarchy can be affected by other subsystems. However, this research develops a prototype of using a systems theoretic approach for the effectiveness analysis within only the middle two-layer subsystems. There are not enough attentions to the effects from other systems; like the organizational context in hospital-level healthcare systems, patients, community, and the role of government, We believe that the basic two pairs concepts of systems theory and the system flow model can be applied to other layers in the healthcare systems. It is hoped that this analysis will stimulate wider debate on the application of holistic systems analysis for improving the effectiveness of systems on quality and safety in health care.

## Competing interests

The authors declare that they have no competing interests.

## Authors' contributions

S.W. Chuang performed the literature review and model development, and drafted the manuscript. K. Inder critically examined the manuscript for intellectual content. All authors read and approved the final manuscript.

## Pre-publication history

The pre-publication history for this paper can be accessed here:


